# A Review of Prebiotics Against *Salmonella* in Poultry: Current and Future Potential for Microbiome Research Applications

**DOI:** 10.3389/fvets.2018.00191

**Published:** 2018-08-15

**Authors:** Andrew C. Micciche, Steven L. Foley, Hilary O. Pavlidis, Donald R. McIntyre, Steven C. Ricke

**Affiliations:** ^1^Department of Food Science, Center for Food Safety, University of Arkansas Fayetteville, AR, United States; ^2^Division of Microbiology, National Center for Toxicological Research, U.S. Food and Drug Administration Jefferson, AR, United States; ^3^Diamond V Cedar Rapids, IA, United States

**Keywords:** prebiotics, *Salmonella*, poultry, microbiomics, metabolomics, fructooligosaccharides, mannanoligosaccharides, galactooligosaccharides

## Abstract

Prebiotics are typically fermentable feed additives that can directly or indirectly support a healthy intestinal microbiota. Prebiotics have gained increasing attention in the poultry industry as wariness toward antibiotic use has grown in the face of foodborne pathogen drug resistance. Their potential as feed additives to improve growth, promote beneficial gastrointestinal microbiota, and reduce human-associated pathogens, has been well documented. However, their mechanisms remain relatively unknown. Prebiotics increasing short chain fatty acid (SCFA) production in the cecum have long since been considered a potential source for pathogen reduction. It has been previously concluded that prebiotics can improve the safety of poultry products by promoting the overall health and well-being of the bird as well as provide for an intestinal environment that is unfavorable for foodborne pathogens such as *Salmonella*. To better understand the precise benefit conferred by several prebiotics, “omic” technologies have been suggested and utilized. The data acquired from emerging technologies of microbiomics and metabolomics may be able to generate a more comprehensive detailed understanding of the microbiota and metabolome in the poultry gastrointestinal tract. This understanding, in turn, may allow for improved administration and optimization of prebiotics to prevent foodborne illness as well as elucidate unknown mechanisms of prebiotic actions. This review explores the use of prebiotics in poultry, their impact on gut *Salmonella* populations, and how utilization of next-generation technologies can elucidate the underlying mechanisms of prebiotics as feed additives.

## Introduction

*Salmonella* can be spread through the fecal-oral route (1, 2), and is a concern for pathogenic contamination of poultry meats and eggs used for human consumption. Previously this concern had been mitigated through the use of antibiotics, which also promoted animal growth (3). However, with the rise of multidrug-resistant bacteria (4–6), the food industry has been pursuing alternative control measures for pathogenic *Salmonella* contamination. These approaches include but are not limited to chemical-based interventions, such as organic acids and essential oils, or biological-based treatments, such as bacteriophage, probiotic, and prebiotic therapies.

The recent use of prebiotics has been well documented. The term “prebiotic” was first coined by Gibson and Roberfroid in 1995 and defined as “a nondigestible food ingredient that beneficially affects the host by selectively stimulating the growth and/or activity of one or a limited number of bacteria in the colon, and thus improves host health” (7). Gibson and Roberfroid (8) demonstrated that the intake of prebiotics could regulate specific gastrointestinal tract (GIT) microorganisms to alter the microbiome. Over the years, further findings have led to several suggested modifications of the definition such as the addition of the term “selectively fermentable” (9) or the term “nonviable” (10, 11). More recently, an expert consensus from the International Scientific Association for Probiotics and Prebiotics (ISAPP) defined prebiotics as “a substrate that is selectively utilized by host microorganisms conferring a health benefit” (12).

Prebiotics have been used to influence the growth of reported beneficial bacteria in the GIT, such as *Bacteroides* and *Bifidobacterium* (13–16). Van Loo et al. (17) detailed several natural sources of prebiotics including garlic, onions, and asparagus. Typically including fiber and oligosaccharides (18), prebiotics in chickens increase amylase production in the GIT and therefore improve the overall growth rate of broilers (16). They reduce colonization of *Salmonella* during hen molting (19). Some prebiotics have also influenced protection against *Salmonella* by providing binding sites for bacteria to be flushed out of the digestive tract (18). Numerous studies have also seen the reduction of *Salmonella* populations by increasing short chain fatty acids (SCFAs) concentrations (20–22) which can be accomplished through prebiotic administration (23, 24).

Furthermore, several studies (25–29) investigated prebiotic effects on the GIT microbiota through 16S microbiome sequencing. By also noting changes in metabolite concentrations or metabolomics, this approach may be able to correlate changes in the microbiome to changes in the metabolite concentration such as SCFAs and other, possibly unknown, metabolites that can stymie *Salmonella* growth. The scope of this paper to provide an overview of the literature linking the use of prebiotics to the overall reduction in the number of foodborne *Salmonella* and the repression of virulence factors. The scope of this paper will not detail the other benefits of prebiotics in poultry such as impact on growth performance or antioxidant capacity, as they are covered extensively in Dhama et al. (30, 31), Yadav et al. (32), and other literature reviews. By investigating SCFA production, microbiomic, and metabolomic technologies, and currently utilized prebiotics, notably oligosaccharides, this review attempts to elucidate novel avenues of research into the reduction of virulent pathogens via prebiotics, which may improve the safety of the poultry industry and improve the overall public health by reducing the incidence and or severity of poultry-acquired salmonellosis.

## The poultry gastrointestinal tract

The gastrointestinal tract of chickens is complex due to the bird's large energy requirements (33). The chicken GIT includes the crop, gizzard, duodenum, ileum, and cecum, which are microbiologically abundant with over 900 documented bacterial species (34). Included in the upper segment of the GIT, is the crop, which is used for fermentation, hydrolysis of starch to sugar, food storage, and as an acid barrier with a pH of ~4.5. The gizzard grinds food particles in a highly acidic environment (pH 2.6) (35–38). While the mean retention time throughout the GIT is ~6 h, feed can remain in the crop and gizzard for as little as 8 and 50 min, respectively (39). The crop contains numerous anaerobic bacteria attached to the epithelium, including *Lactobacillus*, and they produce SCFA's and lactic acid (40, 41). The continuous layer of *Lactobacillus*, enterococci, coliforms, and yeast promote digestion of most carbohydrates, with the remainder digested in the ceca after passage through the lower GIT (37, 42).

Lower in the GIT is the duodenum, ileum, and cecum. Digestive enzymes and bile from the pancreas and gallbladder are added to the duodenum to break down food further, allowing for better absorption into the bloodstream through the villi (43). This process is continued through the ileum in the lower small intestine (43). The small intestine is dominated by anaerobic bacteria (44), and contains *Lactobacillus* and *Bifidobacterium* species in high concentrations as well as *Enterococcus faecium* and *Pediococcus* spp. (35, 45, 46). However, despite the presence of these bacteria in the small intestine, the concentrations of bacteria in the ceca are reported to be the highest in the chicken GIT, at ~10^11^ bacteria/g (35, 47, 48).

The ceca are located where the small and large intestines meet, and while they serve no identifiable purpose for digestion in mammals, it is important in chickens for fermentation and overall animal health (33, 35, 43). Due to culturing poultry cecal microbiota on arabinoxylan, it has been suggested the cecum may be involved in the breakdown of grains (42). The cecum plays additional roles in water adsorption and urea recycling, although the full nutritional significance remains unclear (49, 50). However, despite its importance, in an experiment involving ligation of the cecum, it was shown that while nitrogen availability was disturbed by a cecectomy, it was not necessary for survival (51, 52). The ceca, from a food safety standpoint, is also of major significance because it is one of the leading sites for *Salmonella* colonization along with the crop (53–55).

*Salmonella* can be found in varying concentrations in all regions of the poultry GIT of challenged chickens (56, 57). In Fanelli et al. (56), 1 day after the birds were challenged with *Salmonella*, the duodenum and the small intestines were examined, and 5–45% of the samples tested positive depending on the region viewed. However, cecal samples in this study were nearly 100% positive for *Salmonella* colonization (56). This trend continued throughout the 13-day trial. Additional studies found that, when challenged with a lower concentration, *Salmonella* was not recoverable from the duodenum and small intestine despite being isolated from the crop, because bacteria were often destroyed in passaging through the acid lumen of the proventriculus and gizzard (58). While other studies have focused on the crop and even the gizzard as colonization sites of *Salmonella*, the ceca remain the most commonly investigated section of GIT for *Salmonella* (39, 55, 58, 59). This is likely because of the relatively high bacterial counts of up to 10^11^ cells/g of digesta by the day three post-hatch (35, 60). Other reasons may include the ceca being the environment in the GIT most advantageous for *Salmonella* to colonize (56), and because the ceca can be ruptured during processing. However, it should be noted, Hargis et al (55) found that crops was 86 fold more likely to rupture than ceca during processing. Despite this focus on the ceca, with the potential for each organ's microbial composition to influence the next downstream, it is vital to understand the microbiota of each region of the avian GIT.

Stanley et al. (35) compiled data from several papers detailing the most prevalent microbial groups in each of the GIT regions. They found that while *Lactobacillus* was prominent, if not dominant in all systems, a myriad of differences was reported, including *Clostridiaceae* and *Enterococcus* in the crop and gizzard, and that a majority of cecal bacteria were not culturable or described. However, these profiles can vary greatly, as it has been suggested that host genotype, sex, and age play an important role in determining microbial composition (61). Furthermore, a majority of the collected papers reported information using community-fingerprinting techniques such as temporal temperature gradient electrophoresis (TTGE) and terminal-restriction fragment length polymorphism (T-RFLP), as well as culture-based methods. These techniques provide useful information, such as the application of T-RFLP in Torok et al. (25), which helped identify the presence of over 600 bacteria species and 100 distinct genera in the GIT of chickens. However, each of these techniques exhibits significant issues. Community fingerprinting techniques in general, are considered only semi-quantitative and are only capable of detecting taxa in abundance of >1% (61, 62). Additionally, culture-dependent methods are particularly limited. For example, in the cecum, only 10–60% of bacterial strains have been cultured (63, 64). Therefore, while these techniques have generated valuable information, to accurately detail the complex and minute changes to the microbiota under the effect of prebiotics, further investigation with more sensitive methodologies is needed. The changes, however, often depend on the type of prebiotic utilized.

## Commonly used prebiotics

**Figure 1:**
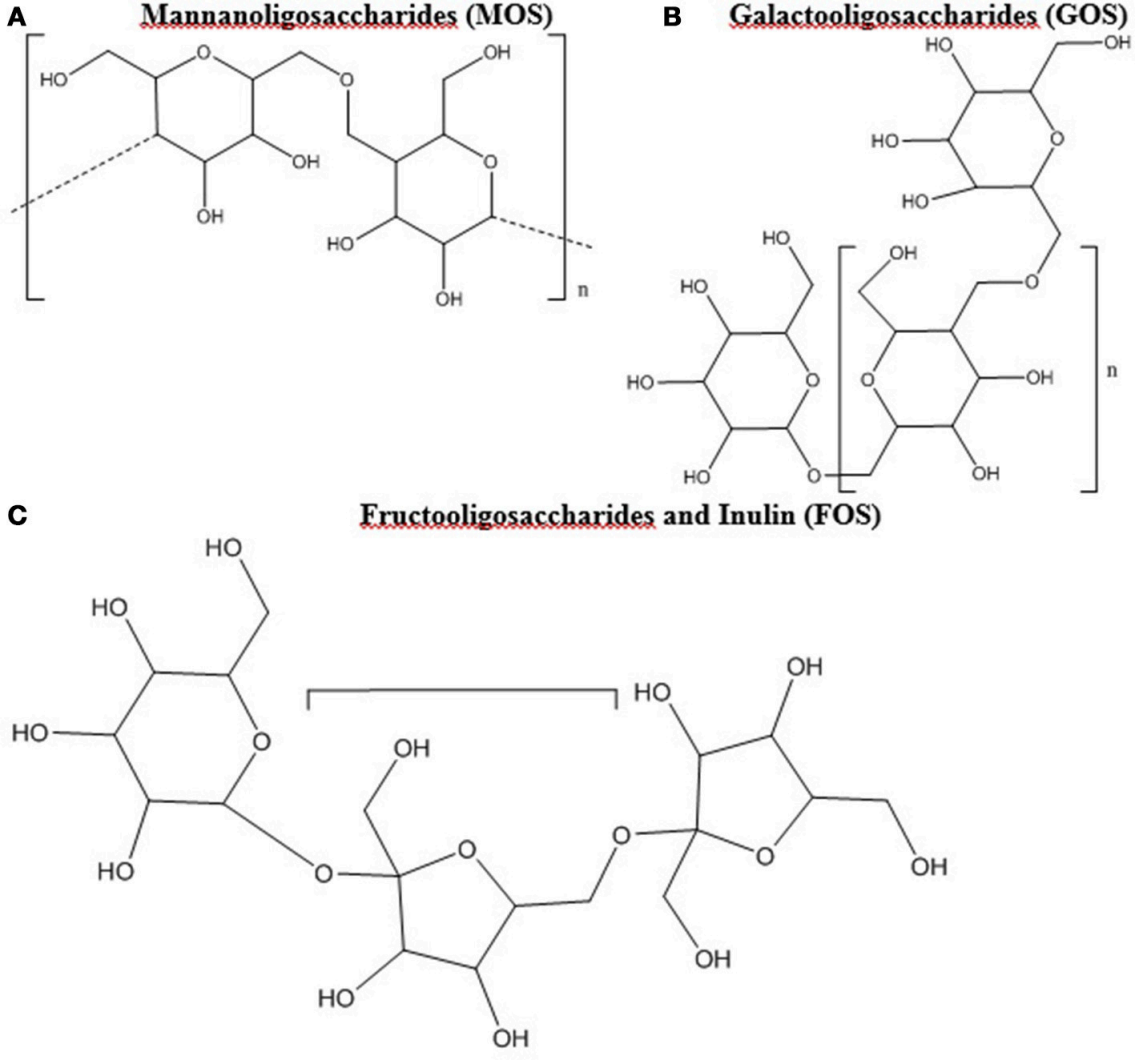
**(A–C)** Chemical Structure of oligosaccharides. All chemical structures were drawn in ChemBioDraw Ultra (PerkenElmer, Waltham, MA).

Prebiotic studies have focused largely on oligosaccharides such as mannanoligosaccharides (MOS), galactooligosaccharides (GOS), and fructooligosaccharides (FOS) including inulin (12, 24, 65–67). Oligosaccharides are polymer chains with 3 to 10 of simple sugars (Figure 1) (68). Oligosaccharides and fiber have been combined and amended with feed products to create commercially viable sources of prebiotics in the poultry industry with a range of results. Illustrations of the modes of action of prebiotics within poultry can be found in Yadav et al. (32) and Pourabedin and Zhao (67).

Several commercial prebiotics have been studied and utilized, such as Biolex® MB40 and Leiber® ExCel (Leiber, Hafenstraße 24, Germany), which are brewer's yeast cell walls composed of MOS (27–29, 69). These products were found to reduce *Campylobacter* concentrations and alter the microbiome, and there is an expectation of MOS-based products to reduce pathogens that utilize mannose-specific type 1 fimbriae such as *Salmonella* (28, 70). Furthermore, Lee et al. (71) did evaluate the effect of these products against *Salmonella* in commercially raised broilers, and while a lower prevalence was noted, only 10 samples were utilized, and a challenge study was not performed. As another example, the commercialized yeast-fermentate product XPC (Diamond V, Cedar Rapids, IA), has reduced *Salmonella* in chickens and increase butyrate in the GIT (27, 29, 72–74). Furthermore, during a *Salmonella* challenge experiment, the addition of XPC, which is comprised of 25% fiber, to chicken feed decreased the expression of virulence factor *hilA*, which is a regulator and promoter within a pathogenicity island (SPI-1) (72, 74). These findings imply that XPC may reduce *Salmonella* virulence and invasion.

While these effects are detectable, synergistic effects can also be created by combining probiotics and prebiotics to create synbiotics. Probiotic products such as All-Lac® have been used in conjunction with Bio-MOS® to alter the microbiome, whereas Fasttrack® (Fasttrack, Conklin, Kansas City MO) and PoultryStar® (PoultryStar, BIOMIN GmbH, Herzogenburg, Austria), contain FOS and have been shown to reduce *Salmonella* and improve feed conversion efficiency (65, 75–77). These products, along with numerous others, have been found to improve poultry GIT health, increase animal weight, and inhibit *Salmonella* and *Campylobacter*. As a consequence, because of the range of available prebiotic products, methodologies of application, and the yield of numerous and sometimes inconsistent results (24, 78, 79), it is vital to understand these prebiotics better. Moreover, it is essential to detail their currently elucidated or suggested mechanisms to refine further ways to improve poultry health and production practices. To capture the effects of the breadth of prebiotics available, several types of prebiotics and their impact on *Salmonella* in poultry will be discussed in this section.

Mannanoligosaccharides (Figure 1A) are found in the cell wall of numerous fungal species including brewer's yeast (*Saccharomyces cerevisiae*) and *Saccharomyces boulardii*, as well as certain plants (67, 66). Comprised of mannose oligomers linked via β-1,4 glycosidic bonds, MOS have been demonstrated to suppress enteric pathogens and enhance the poultry immune system (80). Broiler chickens do not possess enzymes to break down MOS, as such it is suggested that bacteria in the lower GIT, such as the ceca, are responsible for their digestion (67). One particular advantage of MOS as a prebiotic is its stability as a pellet during steaming, which allows it to be easily added to feed (66). Studies have shown that *Salmonella* possessing type 1 fimbriae can be sensitive to the presence of MOS, which can disrupt attachment and adhesion from the intestinal lining by encouraging attachment to the mannose in the lumen (69, 81). The disruption of attachment and adhesion was reported for 53% of tested *Salmonella* strains (81, 82). However not every *S*. Typhimurium strain possesses type 1 fimbriae, as out of 13 tested strains by Mirelmann et al. (83), only 4 expressed type 1 fimbriae.

Mannanoligosaccharides have also been reported to improve overall gut health through increasing villi length and providing an adjuvant-like effect by acting as a microbial antigen (66, 84, 85). One study in particular exhibited a reduction in *Salmonella* ceca population by day 10 in challenged chicks fed a diet consisting of (0.40%) MOS (86). Stanley et al. (87) also demonstrated a one to three log reduction of cecal *Salmonella* counts in 21-day old chicks when supplemented with 0.05% MOS and MgSO_4_. A meta-analysis, which was designed to increase power by combining results from multiple studies, was performed by Hooge (66), which indicated MOS addition to feed generated improved body weight, feed conversion ratios, and survivability. This meta-analysis listed seven selection criteria including date of publication and age of bird and consisted of 29 pen trials from separate studies that were analyzed using a paired *T*-test. However, some discrepancies were noted in MOS ability to improve beneficial microorganisms (80), and there was no set standardization among studies involving the administration of the amount of the prebiotic.

Fructooligosaccharides (Figure 1C) are naturally occurring, typically of plant origin, contain β-(2,1) linkages, and can be food ingredients, functional foods, and prebiotics (8, 88). Due to the β-(2,1)-linkages, enzymatic degradation is difficult in the upper GIT, leading to primary breakdown occurring in the ceca (8, 24, 89). Fructooligosaccharides support the growth of *Lactobacillus* and *Bifidobacterium*, resulting in an increase in SCFAs and lactate, an enhancement of the immune system, and the reduction of *Salmonella* colonization (23, 24, 90, 91). The elucidated mechanism of action for many of these benefits is that FOS is fermented by *Lactobacillus* and *Bifidobacterium* which increases SCFAs and lactate in the cecum resulting in lower *Salmonella* colonization (23, 24). The ability to ferment FOS is present in most strains of *Lactobacillus* and *Bifidobacterium* (24, 92, 93). However, only 8 of 55 strains tested by Rossi et al. (94) were capable of using inulin, which is a long chain FOS derivative, as the sole carbon source.

Furthermore, it was suggested that adverse consequences might exist with the implementation of FOS in poultry feed. Ten Bruggencate et al. (95) demonstrated, in rats, a decrease in *Salmonella* resistance occurred due to an increase in intestinal permeability. Additionally, SCFAs may lead to an enhanced expression of *Salmonella* virulence genes despite reductions in colonization (20, 96). However, inulin amended diets have yielded middling results with Rehman et al. (93) demonstrating that inulin supplementation did not significantly impact the microbial community of the chicken cecum and Ramnani et al. (97) showed no impact on SCFA production in human diets supplemented with inulin. The effectiveness of FOS and inulin is dependent on a number of factors including the composition of the basal diet, degree of FOS polymerization, the presence of *Bifidobacteria* strains, host animal characteristics, and even host stress factors (91, 98). The FOS amended diets in poultry studies have appeared to yield inconclusive results; however, it has been demonstrated that FOS, when supplemented with probiotics, can produce consistently significant reductions in *Salmonella* (24, 79). This potential synergism has led to its implementation in products such as PoultryStar™ that directly impact aspects of the GIT (99, 76).

Galactooligosaccharides (Figure 1B) can be naturally found in human and cow milk, and consist of β-(1,6) and β-(1,4) linkages that avoid digestion in the upper GIT (100–103). Commercially, GOS can be prepared through hydrolyzing lactose from cow's milk and often commercial products contain lactose and a myriad of GOS oligomers (104, 105, 106). For instance, Bimuno (Clasado Ltd) is composed of varying concentrations of lactose and di-, tri-, tetra-, and pentose oligomers of GOS (104, 107, 106). Bimuno, *in vitro* and in mice ileal gut loops, caused reduction of *S*. Typhimurium adhesion and invasion, and but not when GOS was removed from the Bimuno mixture (107). Despite these positive effects, no significant differences in *Salmonella* concentrations was found when poultry was provided feed amended with 1% GOS, although significant alterations to the cecal microbiome were observed (108).

Despite this contrast, while GOS has not been as well studied in poultry compared to FOS and MOS (67), several publications have suggested some potential for GOS as a prebiotic in poultry. A bifidogenic effect has been observed by showing increased counts of *Bifidobacterium* in feces of birds fed 3 g of GOS per 25 kg of feed for 40 days (100). The addition of GOS to feed has also been shown to increase the *Lactobacillus* population in cecal contents (109), and when compared to xylooligosaccharides (XOS), FOS, and MOS, GOS significantly improved *L. reuteri* growth on minimal media (110). Besides promoting the growth of *Bifidobacterium* and *Lactobacillus*, GOS has demonstrated other potentially beneficial effects such as reducing heat stress in the jejunum, but not the ileum (111). GOS has been demonstrated to significantly alter the poultry transcriptome when injected *in ovo* compared to the addition of inulin and *Lactococcus lactis* (112), and also improve cell-mediated immunity when in low concentrations (0.1%) (109).

Additionally, GOS has been utilized as part of a synbiotic in some studies. Synbiotics are defined as a combination of probiotics and prebiotics (113). When *Bifidobacterium* was added to poultry feed along with GOS, this synbiotic affected total anaerobic microbial populations in feces, increasing them from 9.71 to 10.26 log colony forming units per gram (CFU/g) (100). This addition also increased *Lactobacillus* and *Bifidobacterium* fecal counts by 0.53 log and 1.32 log units, respectively (100). When injected *in ovo*, commercialized GOS and *Lactococcus lactis* elevated the body weight of broilers at the end of the rearing period (102, 113). This data differed from Biggs et al. (114) which used only the prebiotic, and by Jung et al. (100) and Abiuso et al. (115), which found no change in body weight when GOS was administered in feed. A cursory examination suggests this variation may be due to the differences in the basal diet and genetic variation of the chickens but more in-depth studies must be performed to ascertain the reason.

Other prebiotics have also been investigated to varying degrees. The implementation of 2 g/kg of XOS increased *Lactobacillus* and acetate in the cecum and after a 5-week treatment, significantly reduced cecal colonization and spleen translocation of *S*. Enteritidis (92, 116). Approximately a one log reduction of *S*. Enteritidis in the cecum was found by Pourabedin et al. (117) when XOS was implemented, but this was lower than the reduction observed by MOS (1.6 log reduction). Additionally, it was found that isomaltooligosaccharides (IMO) improved growth of *Lactobacillus in vitro*, exhibited a bifidogenic effect, and inhibited *Salmonella in vitro* (110, 118, 119). Thitaram et al. (120) found that diets supplemented with 1% IMO could reduce *Salmonella* by a two-log reduction and enhance growth during the first 3 weeks of growth, as well as increasing butyrate concentrations in the jejunum (121).

The effects of dietary fiber has also been investigated and suggested to possess prebiotic properties in poultry (10, 122). Fiber, depending on the derivative, source, and concentration, can accelerate feed passage and can alter the weight of the organs of the poultry GIT in a way that is indicative of improved functioning of the GIT (122–125). Organic acids, such as SCFAs, are a by-product of anaerobic fermentation of dietary fiber, and this suggests the possibility of inhibiting *Salmonella* growth in the GIT (126). As a consequence, there is some discussion if fiber should be considered a prebiotic (10). In Japan, while the term prebiotic is not defined, fiber, along with oligosaccharides are considered “foods to modify the gastrointestinal conditions” and can be considered “foods with specific health uses” (10, 127). Dietary fiber does meet the definition of a prebiotic purported in Gibson et al. (12). However, Roberfroid 128 suggests the need for several additional criteria such as resistance to gastrointestinal absorption, fermentation by intestinal microbiota, and selective stimulation of growth or activity of beneficial bacteria. Under this definition fiber, as well as inulin does not match the criteria for being a prebiotic, despite having some prebiotic effects (46, 128). As such, regulatory agencies such as the FDA and the European Food Safety Authority (EFSA) do not currently consider fiber to be a prebiotic (10, 129).

Regardless of their defined role from a regulatory consideration, there is an apparent variance in the effects these molecules have on the chicken GIT. Due to the complexity of some of these molecules such as fiber, and their effects, to elucidate their mechanisms on *Salmonella* reduction, the changes in the gut microbiota must be observed. To capture these alterations, microbiomic technologies can be employed.

## Microbiomics

With the advent of whole genome and 16S rRNA genomic sequencing, researchers have been able to more accurately quantify microbial population shifts and host responses to the addition of prebiotics (25). By sequencing portions of the highly conserved 16S rRNA gene, such as the V1-V3 or the V4 region, and comparing it to databases, such as the Greengenes database, accurate identification of the microbiome can be determined efficiently and at a relatively lower cost (130, 131).

It should be noted that the rapid advancement in DNA sequencing technologies is continuously allowing for higher throughput at a lower cost (132, 133), and this section will attempt to provide as recent information as possible. Currently, Illumina-based microbiome sequencing can provide Operational Taxonomic Unit (OTU) detection at a very low abundance due to sequencing short DNA strands up to 300 bp. With the Illumina MiSeq Benchtop sequencer (Illumina, San Diego, CA, USA), a three-day sequencing run can return 7.5 Gb from 15 million 300-base paired-end reads to yield bulk data for small-scale projects (132). This efficiency is only increasing as technology allows for faster returns of more substantial data. Large-scale projects to study numerous samples can also use the Illumina HiSeq which allows for parallel sequencing at a comparably lower cost (132). The Illumina HiSeq returns 1,500 Gb from 5 billion 150 base paired-end reads but is typically only considered for production scale laboratory studies (132). Additionally, the Ion Torrent PGM system operates by detecting hydrogen ions that are released during DNA synthesis to sequence the genome israpid and easily scalable (Thermo Fisher Scientific, Waltham, MA, USA) (134–136). To analyze this ever-expanding capacity for bulk genomic data, bioinformatics programs are be employed such as Quantitative Insights Into Microbial Ecology (QIIME) and mothur (131, 137). Despite several differences, such as the programing language utilized, both programs have been shown to compile genomic data and evaluate species richness and equality with little statistical variation (131, 138–141). Using these bioinformatic programs, data can be efficently processed and changes in the GIT microbiome can be elucidated.

Investigative research into prebiotics greatly benefits from the sensitive high throughput technology that can quantitatively measure the differences between testing conditions. Park et al. (26) utilized Illumina based technology and the QIIME pipeline program to assess the changes in the cecal microbiota when subjected to the yeast-based prebiotics, Biolex® MB40, and Leiber® ExCel. They found significant changes in concentrations of *Campylobacteraceae, Faecalibacterium*, and, on the whole, in the phyla *Firmicutes* and *Proteobacteria* (26). This data was supported by Rastall and Gibson (142), and Park et al. (28), which also found an increase in *Faecalibacterium* OTU's during prebiotic treatment and suggested this increase helped facilitate a healthy microbiome, as an increase in *Faecalibacterium* has been linked to health benefits in poultry. Additional investigations into prebiotics found that MOS implementation can significantly alter the bacterial community phylogenetically (143, 144). Park et al. (28) also reported that FOS increased species diversity in pasture flock chickens demonstrated the prominence of *Firmicutes* across all trials, and showed that *Bacteroidetes* decreased in birds fed with diets amended with FOS and GOS. This study also investigated the use of fiber and found it increased the presence of the butyrate-producing *Fusobacterium* (28).

However, these changes only represent broad stroke differences in previously identified major taxa of importance. The aforementioned studies, as well as studies such as Pan (145), have generated not only general information about major taxa shifts but also seemingly negligible differences in the abundance and presence or absence of previously undetailed bacterial strains. While it is important to report changes in previously identified taxa of importance, Illumina sequencing allows for investigation into more nuanced changes or differences found in previously undescribed taxa. For instance, in Park et al. (26), several bacteria that could only be classified to the order *Bacteroidales* were present in chickens fed Biolex® MB40, but were not noted in the control group or birds fed with Leiber® ExCel. These unspecified species may play a potential role in the overall health of the GIT and may have previously gone undetected by culture and community fingerprinting techniques. Some of these nuanced differences can be attributed to variation in individual chicken microbiomes, but, when taken in composite, these data may yield vast and potentially vital information for understanding changes in the avian GIT incurred by prebiotics.

Currently, through analysis of clustered data, it appears the predominant driver of the poultry microbiota composition is host age (28). This deterministic variable was independent of treatments with feeds amended with 1 kg of FOS or plum fibers per ton and 2 kg of GOS per ton (28). While Original XPC™ was able to reduce *Salmonella* cecal populations in Park et al. (27), the microbiota was impacted more by the age of the bird even when in the presence of a coccidiosis vaccine (27, 29). These findings agree with previous assertions regarding the age of the poultry GIT, as it is reported that at birth the GIT is colonized by aerobic organisms followed by anaerobic microbial domination (146). Despite the strong influence of age and other uncontrollable variables such as gender (61), data still indicate that the microbiome can be shifted due to feed amendments. Therefore, because prebiotics can still be utilized to shift the microbial composition of poultry GIT, it is possible to generate environments that are unfavorable for *Salmonella* colonization. This can be accomplished by increasing populations of “healthy” bacteria, preventing space for *Salmonella* colonization as well as increasing SCFA production (67). To understand how these environments can be chemically altered, microbiome technologies can be employed in conjunction with investigative metabolomics technologies.

## Metabolomics

Metabolomics is the qualitative and quantitative identification of all metabolites in a biological system such as the GIT. Metabolites are the final products of cellular processes and can be quantified through a number of instruments such as nuclear magnetic resonance (NMR) and mass spectrometry (MS) (147, 148). Due to its high selectivity, NMR is widely accepted as the primary choice for metabolite elucidation. However, MS is more sensitive comparatively, allowing for detection down to femtomolar (10^−15^) concentrations. Because of this sensitivity, for mixed samples, such as cecal and fecal contents, MS analysis is more readily utilized (147, 149, 150). Mass Spectrometry can also be coupled with chromatography to elucidate the macro-contents of complex mixtures (151). Gas Chromatography (GC) coupled with MS has allowed for the analyses of both volatile and nonvolatile compounds (152). Using GC-MS, Rubinelli et al. (153) investigated the effects of rice bran on *Salmonella* in cecal cultures *in vitro* and detected 578 metabolites. Of these, 367 were unknown, and the change in metabolite concentration was causally linked to the reduction of *Salmonella*. Liquid chromatography has also been used to identify thermolabile molecules in the form of high-pressure liquid chromatography (HPLC) which demonstrated FOS when fed to layers, could reduce cholesterol in eggs (154).

Metagenomic outputs in Sergeant et al. (155) indicated over 200 enzymes that can degrade non-starch polysaccharides in cecal contents, some of which are involved in pathways that produce SCFAs and are vital to the mechanistic understanding of modifying the environment. Unfortunately, one significant drawback to this methodology is the current inability to incorporate genomic information by providing definitive linkages between genotypes and the metabolome (147). Furthermore, the dynamic range of current MS technologies resolving power is ~10^6^, which is far below the estimated concentration of cellular metabolites (147). However, with advances in both high throughput microbiome sequencing and mass spectrometry, it may be possible to derive causal relationships between the presence of phylogenetically related species and concentrations of metabolites.

## Conclusions

The potential for prebiotics to alter the GIT of broiler chickens has been demonstrated with previous generation technologies such as DDGE, T-RFLP, and conventional plating techniques (35). However, despite the success of altering the microbiome, the precise mechanisms, and changes, such as the exact impact of SCFAs on the cecal microbiota, were historically undetermined due to the incomplete analysis offered by the technologies available at the time (156). Furthermore, with a range of variables such as age, type of bird, and genotype, the underlying mechanisms affecting the GIT seemed unlikely to be elucidated. However, with the rising use and affordability of “omic” technologies such as metagenomics and metabolomics, new investigative strategies can be employed. Through the use of bioinformatics pipeline applications on the bulk deep-sequencing data produced by these technologies, there is potential to produce a complete image of the GIT affected by prebiotics. This image may provide predictive power and allow for the understanding and creation, through prebiotics, of an environment that controls for and inhibits *Salmonella* colonization and growth. Moreover, while *Salmonella* is not the only pathogen of concern in the poultry industry, with the potential for virulence gene repression, it is likely prebiotics will continue to play a role in the control of this pathogen. With the ability to utilize next-generation technologies and more fully understand the complexity of the microbiome of poultry GIT, impacts of prebiotics on pathogen control will continue to be elucidated, investigated, and utilized in food safety.

## Author contributions

AM, SF, and SR have made substantial, direct and intellectual contribution to the work, and approved it for publication. HP and DM have been involved in the editing process and approved it for publication.

### Conflict of interest statement

The authors declare that the research was conducted in the absence of any commercial or financial relationships that could be construed as a potential conflict of interest.
